# Ten cases with 46,XX testicular disorder of sex development: single center experience

**DOI:** 10.1590/S1677-5538.IBJU.2016.0505

**Published:** 2017

**Authors:** Emre Can Akinsal, Numan Baydilli, Abdullah Demirtas, Cetin Saatci, Oguz Ekmekcioglu

**Affiliations:** 1 Department of Urology, Erciyes University Medical Faculty, Kayseri, Turkey;; 2 Department of Genetics, Erciyes University Medical Faculty Medical, Kayseri, Turkey

**Keywords:** Chromosome Aberrations, Infertility, 46, XX Testicular Disorders of Sex Development

## Abstract

**Objective:**

To present clinical, chromosomal and hormonal features of ten cases with SRY-positive 46,XX testicular disorder of sex development who were admitted to our infertility clinic.

**Cases and Methods:**

Records of the cases who were admitted to our infertility clinic between 2004 and 2015 were investigated. Ten 46,XX testicular disorder of sex development cases were detected. Clinical, hormonal and chromosomal assessments were analized.

**Results:**

Mean age at diagnosis was 30.4, mean body height was 166.9cm. Hormonal data indicated that the patients had a higher FSH, LH levels, lower TT level and normal E2, PRL levels. Karyotype analysis of all patients confirmed 46,XX karyotype, and FISH analysis showed that SRY gene was positive and translocated to Xp. The AZFa, AZFb and AZFc regions were absent in 8 cases. In one case AZFb and AZFc incomplete deletion and normal AZFa region was present. In the other one all AZF regions were present.

**Conclusion:**

Gonadal development disorders such as SRY-positive 46,XX testicular disorder of sex development can be diagnosed in infertility clinics during infertility work-up. Although these cases had no chance of bearing a child, they should be protected from negative effects of testosterone deficiency by replacement therapies.

## INTRODUCTION

Forty Six,XX testicular disorder of sex development (DSD) is a rare clinical condition with a reported incidence of 1:20.000 in newborn males (1). It was first described by De la Chapelle et al. in 1964 (2) By 1996, only 150 patients with classical 46,XX testicular DSD syndrome have been reported (3); however, more than 100 cases were described in the next ten years (4).

The sex-determining region Y gene (SRY) located in Y chromosome plays a major role in encoding a testis determining factor (TDF) (5, 6). About 90% of 46,XX testicular DSD have Y chromosomal material including the SRY gene, that is usually translocated to the distal tip of the short arm of X chromosome or autosomal chromosomes. About 10% of 46,XX testicular DSD cases are negative for SRY gene, which could carry different degrees of masculinization (4, 7).

Cases with SRY-positive 46,XX testicular DSD are usually diagnosed after puberty when present with hypogonadism, gynecomastia and infertility (1). Short stature and normal mental development are the other clinical characteristics of these patients (4).

In this retrospective study, we analized clinical, chromosomal and hormonal features of ten cases with SRY-positive 46,XX testicular DSD who were admitted to our infertility clinic.

## CASES AND METHODS

Records of the cases who were admitted to our infertility clinic between 2004 and 2015 were investigated. Ten 46,XX testicular DSD cases were detected.

Medical/family history and detailed physical examination records, body mass indexes, presence of parenteral consanguinity, records of each testicular volume measured with Prader orchidometer, serum levels of follicle-stimulating hormone (FSH), luteinizing hormone (LH), estradiol (E2), prolactin (PRL), total testosterone (TT), semen analysis results, karyotype and molecular analysis results and Dual-energy X-ray absorptiometry (DEXA) reports, if present, were assessed retrospectively.

Samples of peripheral blood (3mL) for chromosomal analysis were collected into 0.3mL heparin containing injectors. Conventional method was used on the lymphocyte cultures for karyotype analysis. By G band staining technique 550 level bands were obtained and twenty metaphases were counted.

The probe mix containing SRY gene, Yp11.31 locus specific probe, labelled in red, and control probes for the X centromere (DXZ1), labelled in blue, and for chromosome Y (DYZ1, the heterochromatic block at Yq12), labelled in green which was used for FISH analysis was purchased from Cytocell (Oxford Gene Technology, UK). After the harvest of cultured blood samples and FISH slide preparation, probe mix was applied according to the manufacturer’s instructions, and materials were examined by using the Nikon, ECLIPSE E1000 fluorescent microscope (Tokyo, Japan) and analized with the CytoVision software (CytoVision, AB Imaging, Germany).

Samples of peripheral blood for sequence analysis were collected into commercially available EDTA-treated tubes. DNA was isolated from peripheral blood samples drawn from control, and study groups using High Pure Polymerase chain reaction (PCR) Template Preparation Kit (Roche Applied Science). After amplifying AZF regions by using PCR mixture containing 10xPCR Buffer 5µL, 2.5mM dNTP 3µL, 25mM MgCl 3µL, 10pmol Primer-1 3µL, 10pmol Primer-2 3µL, Taq DNA polymerase 0.5µL, DNA 5µL and completed to total volume of 50µL by adding water. Primers are specific to AZFa (sy81p1, sy81p2, sy82p1, sy82p2, sy84p1, sy84p2), AZFb (sy127p1, sy127p2, sy142p1, sy142p2, sy164p1, sy164p2, rbm1p1, rbm1p2), and AZFc (sy254p1, sy254p2, sy255p1, sy255p2, sy277p1, sy277p2, cdyp1, cdyp2, bpy2p1, bpy2p2) regions and were used separately in mixture. After 40 cycles of PCR by using “94ºC 40 seconds, 58ºC 40 seconds, 72ºC 45 seconds” program, final product was loaded to 2% agarose gel. After electrophoresis, gel was examined by using UV transilluminator for absence or presence, location and, size of bands.

The medical ethics committee of Erciyes University approved this study and informed consent was obtained from all patients.

## RESULTS

In our ten cases, mean age at diagnosis was 30.4, mean body height was 166.9cm (lower than general population), mean body weight was 72.6kg, mean BMI was 26.02. Semen analysis showed azoospermia and average semen volume was 1.68mL. All cases had small testicular volumes. Mean testicular volume was 3.1mL for right testes and 2.5mL for left testes. Detailed general characteristics of the cases are presented in Table-1.

**Table 1:** Clinical data and semen volume analysis.

Cases	Age (years)	Height (cm)	Weight (kg)	BMI (kg/m2)	TV (R/L) (mL)	EV (mL)
1	26	170	63	21.8	6/5	2.4
2	31	167	72	25.8	2/2	2.5
3	28	169	71	24.9	5/1	1.6
4	30	170	72	24.9	2/2	1.1
5	39	161	74	28.5	3/3	2
6	40	168	81	28.7	5/4	2.5
7	30	162	66	25.1	4/3	3.2
8	28	165	68	25	1/1	0.2
9	24	163	66	24.8	1/1	0.1
10	28	174	93	30.7	2/3	1.2
Mean	30.4	166.9	72.6	26.02	3.1/2.5	1.68

**BMI =** Body Mass Index; **TV =** Testicular Volume; **EV =** Ejaculate Volume; **R =** Right; **L =** Left

Two of the patients (patients 3, 10) had prior orchiopexy operation in their medical history and one of them (patient 3) had parental consanguinity. All patients had no family history for genetic disorders.

Four patients (patients 1, 5, 9, 10) had decreased axillary and pubic hair and one patient (patient 5) had gynecomastia Tanner stage III.

Hormonal data indicated that the patients had a higher FSH, LH levels, lower TT level and normal E2, PRL levels (Table-2).

**Table 2:** Hormonal status of the patients.

Cases	FSH (mIU/mL)	LH (mIU/mL)	E2 (pg/mL)	TT (ng/dL)	PRL (ng/mL)
1	58.1	42.2	52.0	183.0	5.6
2	36.0	16.8	57.9	376.0	3.5
3	35.4	9.5	48.6	94.4	8.1
4	17.9	10.3	24.6	272.7	7.9
5	37.5	16.3	24.5	96.6	6.9
6	36.8	9.8	53.8	290.0	5.6
7	57.3	16.9	49.2	153.6	6.4
8	50.5	11.3	39.1	103.0	9.0
9	43.1	17.9	21.2	242.0	8.6
10	31.3	23.5	27.6	203.0	9.0
Mean and 95% CI	40.4 (31.5-49.2)	17.4 (10.4-24.4 )	39.8 ( 29.7-49.9)	201.4 (134.0-268.7)	7.1 (5.8-8.3)

**FSH =** Follicle-stimulating Hormone (1.5 -12.4 mIU/mL); **LH =** Luteinizing Hormone (1.7-8.6 mIU/mL); **E2 =** Estradiole (25.8-60.7 pg/mL); **PRL =** Prolactin (4.0-15.2 ng/mL); **TT =** Total Testosterone (280-800 ng/dL); **95% CI =** Confidence interval of mean References interval of hormones (FSH, LH, E2, PRL, TT) are given in parentheses

Three patients had dual-energy x-ray absorptiometry (DEXA) assessment. First one (Patient 5) was assessed osteopenic for lumbar vertebraes and femoral neck; second one (Patient 8) was assessed osteopenic for lumbar vertebraes and third one (Patient 7) was assessed osteoporotic for lumbar vertebraes and osteopenic for femoral neck.

Initial karyotyping analysis of all patients were considered as 46,XX with some hesitation because of derivative X chromosomes observed in metaphase fields. After fluorescence in situ hybridization (FISH) analysis, it was revealed that all patients were SRY positive. Next step which was the determination of the presence or the deletion of AZFa, AZFb, and AZFc regions revealed that all these regions were deleted in eight patients, in one patient (patient 9) none of the regions were deleted and in one patient (patient 2) AZFb and AZFc regions were deleted and AZFa was present.

**Figure 1:**
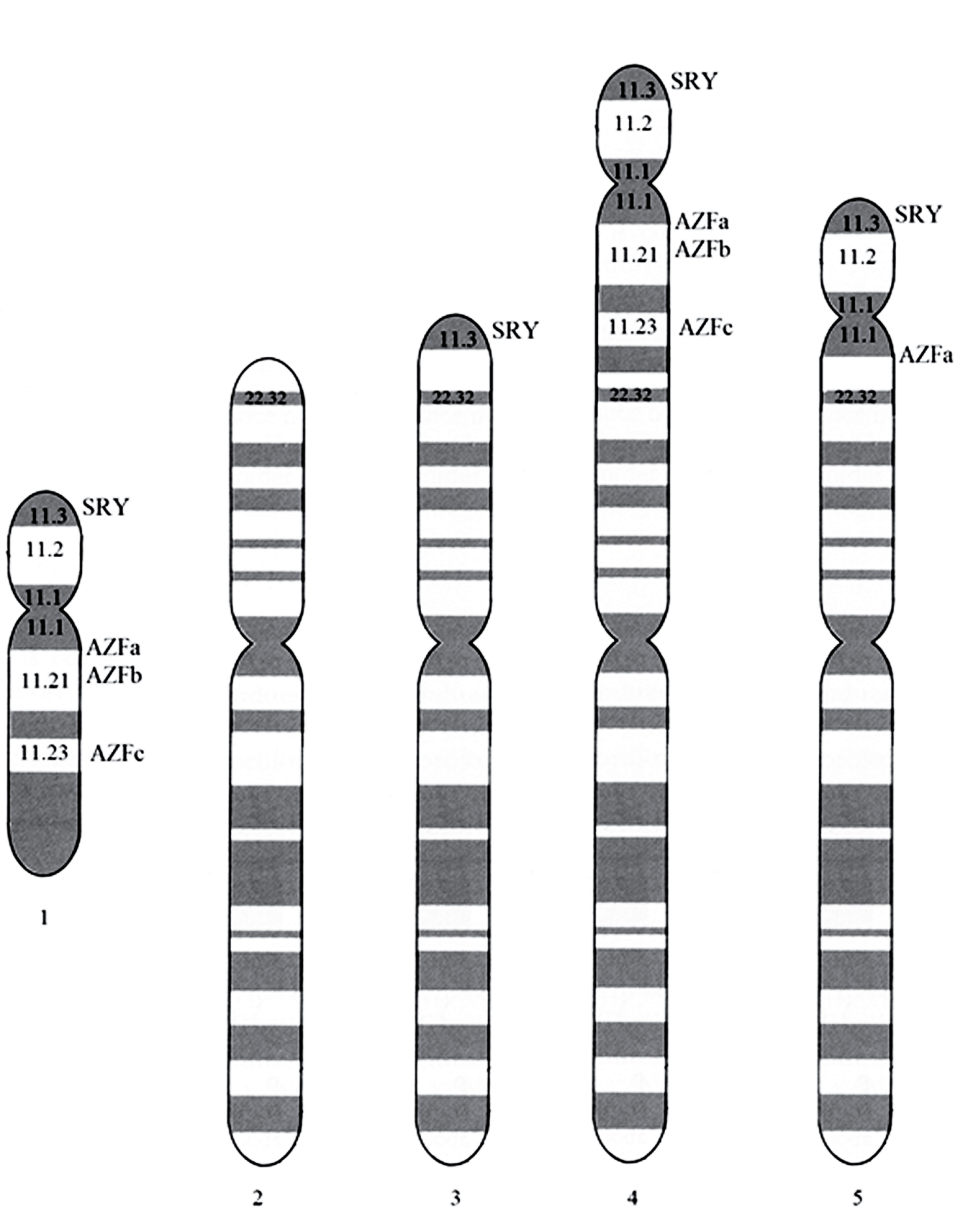
Presumable Ideogram of chromosomes.

Final karyotype for patients was as 46,XX, final FISH report was written as 46,XX ish der (X) t(X;Y) (p22.3;p11.3) (SRY+) and AZF region deletion summarized in Table-3. Presumable Ideogram of the chromosomes is given in Figure-1.

**Table 3:** AZF region analysis of the patients.

Patient	AZFa region	AZFb region	AZFc region
1	-	-	-
2	+	-	-
3	-	-	-
4	-	-	-
5	-	-	-
6	-	-	-
7	-	-	-
8	-	-	-
9	+	+	+
10	-	-	-

- deletion of gene region; + presence of gene region

**1 -** Normal Y Chromosome; **2 -** Normal X Chromosome; **3 -** Derivative X Chromosome of cases number 1, 3-8, and 10; **4 -** Derivative X Chromosome of case 2; **5 -** Derivative X Chromosome of case 9.

## DISCUSSION

46,XX testicular DSD is a rare sex reversal syndrome characterized by a female karyotype in discordance with a male phenotype (4). Although 46,XX male DSD is frequently sporadic (8), familial cases have also been reported (9). All our patients were considered sporadic based on their family history.

46,XX testicular DSD patients can be classified into two groups according to presence or absence of SRY gene (10). Appearance of the external genitalia and masculinization are usually normal in 46,XX SRY-positive testicular DSD cases (4). Before puberty there is no clinical sign except for undescended testis and therefore 46,XX SRY-positive males are usually diagnosed in late adolescence or adulthood through chromosome analyses performed for infertility and/or small testis (11). Two patients had prior orchiopexy operation in their medical history, although, chromosomal analysis is not required for all patients with undescended testes. Possibility of chromosomal abnormality should be kept in mind for azoospermic patients who had prior orchiopexy history. Furthermore, it is a well-known fact that infertile men have 8 to 10 times more chromosomal anomalies than fertile men do, and many times, do not present with other phenotypic characteristics (12). Based on prevalence data, recommendation is made that routine karyotyping be requested of infertile men with deficient spermatogenesis and sperm concentrations lower than 10 million/mL before they are submitted to any assisted reproduction technique (13).

Vorona et al. stated that 46,XX men tend to be shorter than men with Klinefelter syndrome (7). In a previous study, Y chromosome growth-control gene had a possible impact on growth (14). Mean body height was relatively lower than normal population in our cases. Absence of specific growth genes in the Y chromosome may have some effects for this situation.

Classical 46,XX testicular DSD have normal testosterone level and free testosterone level during adolescence, but may decrease in adulthood, leading to hypergonadotropic hypogonadism (15). Testicular volumes are usually lower than 5mL in these cases. While testis morphology is normal in infancy, hyalinization of the seminiferous tubules in early childhood causes loss of spermatogonia (16, 17). Gunes et al. showed hyalinization of the seminiferous tubules by testıcular biopsies of a patient with 46,XX testicular DSD (18). Low testicular volume and hypergonadotropic hypogonadism are constant findings in our patients. But testicular biopsies were not performed because these patients had no chance of bearing a child by assisted reproductive techniques.

Imaging of the pelvis is required to look for remnants of mullerian ducts that may cause morbidity in the form of repeated infections or hematuria and require surgical removal (19, 20). Differential diagnosis with other genetic conditions such as Persistent Mullerian Duct Syndrome in which virilization is achieved despite having low testosterone levels may be a challenging issue in some clinical presentations.

Recent epidemiologic studies have suggested that hypogonadal concentrations of TT are associated with an increased risk of fragility fracture (21, 22). Replacement therapies may protect these patients from bone fracture risk. Prior to initiating therapy, a baseline bone density scan should be performed to look for osteopenia or frank osteoporosis (23). Patients with a T score of <-1.0 would benefit from treatment with Vitamin D and calcium, bisphosphonates, or calcitonin, and require annual repeats of DEXA scan until results are normal (16). Our three patients had DEXA assay and all of them were assessed as osteopenic or osteoporotic. Bone mineral density measurements were absent in the records of our initial patients. We began to perform these measurements and apply replacement therapies as our clinical experience increased. Important part of individuals with SRY-positive 46,XX testicular DSD are diagnosed at adulthood in infertility clinics. But testosterone replacement should be given to patients which have clinical and/or laboratory signs of androgen deficiency in puberty.

During genetic counselling, it was advised to patients that this report must not affect their life and they must continue to live as before. While explaining inheritance it was stated that SRY-positive 46,XX testicular DSD is generally not inherited because of infertility of patients and de novo occurrence of Y and X chromosome translocation. But as there is always possibility of paternal balanced translocation or gonadal mosaicism, chromosome analyses were advised to father and brothers.

In conclusion, chromosomal abnormalities such as SRY-positive 46,XX testicular DSD can be diagnosed in infertility clinics during infertility work-up. Although these patients had no chance of bearing a child, they should be protected from negative effects of testosterone deficiency by replacement therapies.
